# Characterisation of Non-Autoinducing Tropodithietic Acid (TDA) Production from Marine Sponge *Pseudovibrio* Species

**DOI:** 10.3390/md12125960

**Published:** 2014-12-10

**Authors:** Catriona Harrington, F. Jerry Reen, Marlies J. Mooij, Fiona A. Stewart, Jean-Baptiste Chabot, Antonio F. Guerra, Frank O. Glöckner, Kristian F. Nielsen, Lone Gram, Alan D. W. Dobson, Claire Adams, Fergal O’Gara

**Affiliations:** 1 BIOMERIT Research Centre, School of Microbiology, University College Cork—National University of Ireland, Cork, Ireland; E-Mails: catriona.harrington@gmail.com (C.H.); J.Reen@ucc.ie (F.J.R.); marlies.mooij@mumc.nl (M.J.M.); fiona.stewart204@gmail.com (F.A.S.); jeanba-cha@hotmail.fr (J.-B.C.); c.adams@ucc.ie (C.A.); 2 Microbial Genomics and Bioinformatics Research Group, Max Planck Institute for Marine Microbiology, Bremen D-28359, Germany; E-Mails: afernand@mpi-bremen.de (A.F.G.); fog@mpi-bremen.de (F.O.G.); 3 Jacobs University Bremen gGmbH, Bremen 28759, Germany; 4 Department of Systems Biology, Technical University of Denmark, DK-2800 Kgs. Lyngby, Denmark; E-Mails: kfn@bio.dtu.dk (K.F.N.); gram@bio.dtu.dk (L.G.); 5 Environmental Research Institute, University College Cork—National University of Ireland, Cork, Ireland; E-Mail: a.dobson@ucc.ie; 6 Curtin University, School of Biomedical Sciences, Perth, WA 6845, Australia

**Keywords:** TDA, *Pseudovibrio*, marine, antimicrobial, clinical

## Abstract

The search for new antimicrobial compounds has gained added momentum in recent years, paralleled by the exponential rise in resistance to most known classes of current antibiotics. While modifications of existing drugs have brought some limited clinical success, there remains a critical need for new classes of antimicrobial compound to which key clinical pathogens will be naive. This has provided the context and impetus to marine biodiscovery programmes that seek to isolate and characterize new activities from the aquatic ecosystem. One new antibiotic to emerge from these initiatives is the antibacterial compound tropodithietic acid (TDA). The aim of this study was to provide insight into the bioactivity of and the factors governing the production of TDA in marine *Pseudovibrio* isolates from a collection of marine sponges. The TDA produced by these *Pseudovibrio* isolates exhibited potent antimicrobial activity against a broad spectrum of clinical pathogens, while TDA tolerance was frequent in non-TDA producing marine isolates. Comparative genomics analysis suggested a high degree of conservation among the *tda* biosynthetic clusters while expression studies revealed coordinated regulation of TDA synthesis upon transition from log to stationary phase growth, which was not induced by TDA itself or by the presence of the C10-acyl homoserine lactone quorum sensing signal molecule.

## 1. Introduction

Due to the misuse and overuse of antibiotics, the emergence of pathogens that are resistant to virtually all of the currently available antibiotics has reached a critical stage. For this reason, there has been an urgent drive towards the discovery of new antimicrobial compounds. The marine environment provides a relatively untapped source of these unique natural products. In particular, marine sponges are emerging as a goldmine of antimicrobial activity, with many potential bioactive compounds produced by symbiotic bacteria associated with marine sponges [1]. As sessile organisms, sponges rely on an arsenal of metabolites, generally produced by their associated microorganisms, to defend against disease and to gain a competitive advantage within the marine ecosystem [2]. This symbiotic relationship is essential for sponge efficiency and survival [3]. Consequently, more novel bioactive metabolites are retrieved from sponges each year than any other marine organism [2], with approximately 3500 novel marine compounds isolated from marine sponges since 1985 [4]. Recent evidence suggests that the majority of bioactive compounds isolated from sponges are likely to be produced by associated microbiota [5].

*Pseudovibrio* species are ubiquitous in the marine environment, and in particular within marine sponges. They were first isolated and described in 2004 from seawater in Taiwan [6] but have since been isolated from ascidians [7] tunicates [8], algae [9], coral [10], tube worms [11] and from a plethora of marine sponges [12,13,14,15,16,17]. *Pseudovibrio* is in fact believed to be one of the few confirmed vertically transmitted sponge symbionts, due to its association with sponge larvae [18]. Antimicrobial activity within *Pseudovibrio* sp. has been documented a number of times [12,15,16,17,19,20]. Moreover, *Pseudovibrio* species produce specific bioactive compounds, such as heptylprodigiosin [20] and biosurfactant compounds [11]. More recently, the antibacterial compound tropodithietic acid (TDA) was recovered from *Pseudovibrio* sp. D323, isolated from the red alga *Delisea pulchra* [9], which correlated with the previous prediction of TDA production by *Pseudovibrio* *sp*. strain JEO62 based on the presence of the *tdaA-tdaF* biosynthetic cluster in its genome [5,21]. TDA was initially isolated from several species within the *Roseobacter* clade including *Phaeobacter inhibens* and *Ruegeria* sp. [21,22,23,24]. TDA is a suphur containing compound with a unique structure consisting of a dithiet moiety fused to tropone-2-carboxylic acid, which is believed to co-exist with its tautomer, thiotorpocin [25], previously identified in *Pseudomonas* sp. [26,27]. TDA has been shown to have a strong inhibitory activity against a range of marine bacteria, such as *Proteobacteria*, *Actinobacteria*, *Firmicutes* and *Bacteroidetes*, the fish pathogens *Vibrio anguillarum* and *Vibrio splendidus* [28] as well as marine algae [21] and a range of human pathogenic bacteria [24]. It has been proposed as a potential fish larval probiotic, as it has been shown not only to reduce mortality in the larvae of fish such as turbot and cod [29], but also that it has no toxic effects against the eukaryotic models *C. elegans* and *Artemia* sp. [30].

Here, we identify TDA production in *Pseudovibrio* species isolated from the marine sponges *Axinella dissimilis*, *Polymastia boletiformis* and *Haliclona simulans* and investigate phenotypic characteristics, bioactivity and molecular mechanisms of its production.

## 2. Results and Discussion

### 2.1. Culture Conditions Enhance the Production of Bioactive Compounds by Pseudovibrio Species

**Figure 1:**
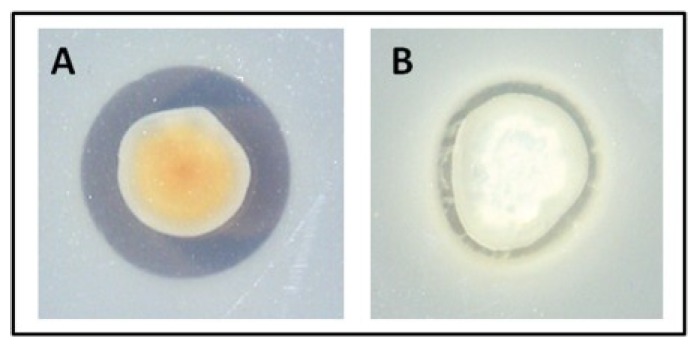
Overlay assay with tropodithietic acid (TDA) producing *Pseudovibrio* isolate WC22—representative of most *Pseudovibrio* isolates. *S. aureus* NCDO949 was used as an indicator strain and was strongly inhibited when the *Pseudovibrio* colony had turned brown. (**a**) Marine agar; and (**b**) starch-yeast extract-peptone–sea water (SYP-SW)_agar.

A collection of 72 *Pseudovibrio* isolates from the marine sponges *Axinella dissimilis*, *Polymastia boletiformis* and *Haliclona simulans*, which were previously classified into 33 groups based on their RAPD profiles [31], was further investigated for antimicrobial activity. A representative strain from each RAPD group was tested for bioactivity via a spot-plate overlay assay on both SYP-SW and marine (MA) agar, against the indicator strain *S. aureus* NCDO 949. Zones of inhibition were produced by 26 of the 33 representatives of the RAPD groups on SYP-SW. Interestingly, all these strains displayed higher levels of inhibition when grown on MA than on SYP-SW (Figure 1), while 5 strains displayed antimicrobial bioactivity only when grown on MA. Two strains displayed no bioactivity against *S. aureus* NCDO 949 on either media (Supplementary Table S1).

In order to establish the spectrum of bioactivity produced by the marine isolates, each individual isolate was tested against a range of clinical and fish pathogens, as well as a number of laboratory strains (Supplementary Table S2). High levels of inhibition were seen in 31 of the 33 strains against both clinical and fish pathogens (Table 1). The vast majority of the isolates displayed antibacterial activity against *V. anguillarum* (88%), *E. tarda* (88%), *Y. ruckerri* (94%), *E. coli* MUH (82%), *E. coli* NCIMB (85%), *M. morganii* (88%), *S. Typhimurium* LT2 (52%), *S. Typhimurium* C5369 (76%), *P. sputonum* (64%), *S. arizonae* (67%) and *S. aureus* NCDO 949 (94%). Interestingly, each of the 31 isolates showing antimicrobial bioactivity produced a characteristic brown pigment when grown on MA. This brown pigment was not produced by the two strains which showed no inhibitory activity. This strongly suggested a direct association between pigment production and the antimicrobial bioactivity. Four *Pseudovibrio* isolates which produced the brown pigment and displayed particularly potent levels of bioactivity—namely W64, W69, W74 and W89—were selected for further analysis.

**Table 1:** Antimicrobial activity of the 33 RAPD group representatives against a range of human and fish pathogens.

Strain	Antagonistic Activity against Pathogen										
	*Y. ruckerri*	*E. tarda*	*V. anguillarum*	*E. coli* MUH	*E. coli* NCIMB 949	*M. morganii*	*S.* Typhimurium LT2	*S.* Typhimurium C5369	*P. sporunum*	*S. arizonae*	*S. aureus* NCDO 949
**JIC5**	+++	+++	+++	++	+	+++	-	++ *	+ *	+ *	++
**JIC6**	+	++	+	-	+ *	+ *	-	-	+ *	-	++
**JIC17**	+++	++	+	-	+ *	++	+ *	-	+ *	+ *	+
**W10**	+++	++	-	+	-	+ *	-	+*	+ *	-	+
**W19**	-	-	-	-	-	-	-	-	-	-	-
**W62**	+++	++	++	+	+ *	+++	-	+ *	++ *	-	++
**W63**	+++	+++	-	-	-	-	-	++ *	+++ *	+ *	++
**W64**	+++	+++	+++	+	+	+++	++ *	++ *	++	++	++
**W65**	+++	++	+++	++	+	+++	+ *	++ *	+ *	+	++
**W69**	+++	+++	++	+	+	++	++ *	++ *	++ *	++ *	++
**W71**	+++	++	++	+	+	+++	+ *	++ *	+ *	+	+
**W89**	+++	+++	+++	+	++	++	++*	++ *	++ *	+	++
**W99**	+++	+++	+++	++	+	+++	+ *	++ *	+ *	+	++
**WC43**	++	-	++	+ *	+	+ *	+ *	+ *	+ *	+	++
**W74**	+++	++	+	+	++	+++	+ *	++ *	+ *	++ *	+
**W85**	+++	++	+++	++	+ *	+	+ *	+ *	+ *	-	++
**W78**	++	+	++	+ *	+ *	+++	+*	+ *	+ *	-	+
**W94**	++	+	++	+ *	+ *	++	+ *	+ *	+ *	+ *	++
**W96**	+++	++	++	+ *	+ *	++	+ *	++ *	-	++ *	+
**WM31**	+++	++	+++	+ *	+ *	+	+ *	+ *	-	-	++
**WM33**	++	+	+	+ *	+ *	-	+	-	-	-	+
**WM34**	+++	++	+++	+ *	+ *	+++	+ *	+ *	+ *	+ *	++
**WM40**	+++	++	++	+	+	++	-	++	++ *	++ *	++
**WM50**	-	-	-	-	-	-	-	-	-	-	-
**WC13**	+++	++	++	+	+ *	++	-	+ *	-	+ *	++
**WC15**	+	-	+ *	-	-	+ *	-	-	-	-	+
**WC21**	++	+ *	++	+	+ *	++	-	+ *	-	+ *	++
**WC22**	+++	+	+	+	+ *	+	-	-	-	+ *	++
**WC30**	+++	+	++	+	+ *	+	-	+ *	-	+ *	+
**WC32**	+++	++	++	++	+ *	++	-	++ *	+ *	+ *	++
**WC41**	+++	++	++	+	+ *	++	-	++ *	-	++ *	++
**HC6**	+++	++	+++	++ *	+	+++	+ *	++ *	+ *	+ *	++
**HMMA3**	++	+	++	+ *	+ *	+ *	+ *	-	-	-	+

Diameter of growth inhibition (mm): + ≥1 mm; ++ ≥2 mm; +++ ≥4 mm. * partial inhibition. Blue: fish pathogen, yellow: laboratory strain, red: human pathogen.

### 2.2. Pseudovibrio Species Derived from Marine Sponge Produce Tropodithietic Acid (TDA)

**Figure 2:**
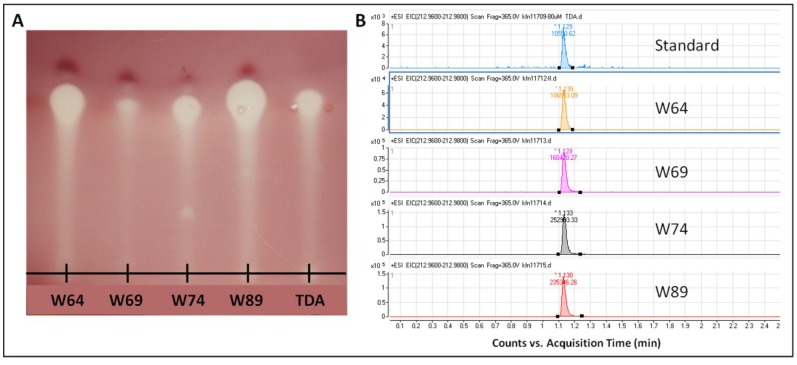
(**a**) TLC-overlay assay of extracts from Pseudovibrio isolates. *S. aureus* NCD0 949 was used as an indicator strain. The concentration of the TDA control was 0.01 mg/mL; (**b**) UHPLC-DAD-qTOFMS data, showing the extracted ion chromatograms (212.9674 ± 0.005) of the [M + H]^+^ ion of TDA. Analysis demonstrated that the bioactive compound produced by 4 *Pseudovibrio* isolates was tropodithietic acid.

Previously the production of a brown pigment associated with antimicrobial activity by Tropodithietic Acid (TDA) producing marine isolates on MA has been reported [28,32], and high levels of activity of TDA against fish pathogens has been documented a number of times [23,30,33] as has activity against human pathogens [24]. While chemical analysis of the pigment has yet to be reported, it has been shown to rely on the same biosynthetic genes as TDA itself, suggesting a direct link to the antimicrobial compound [34]. Thus, the pigmented antimicrobial activity of the *Pseudovibrio* isolates may be due at least in part to TDA. To establish that the *Pseudovibrio* isolates were indeed producing TDA, a method adapted from Porsby *et al.* [24] for the isolation of TDA was employed to extract compounds from the four *Pseudovibrio* isolates with high levels of antimicrobial activity. TLC was performed on the *Pseudovibrio* extracts and plates were overlaid with *S. aureus* NCDO 949 and tetrazolium salt. A large zone of inhibition was observed at the position correlating to an Rf value of 0.71, the same as the TDA control, suggesting that the compound produced by the *Pseudovibrio* isolates W64, W69, W74 and W89 was the secondary metabolite TDA (Figure 2a).

To further confirm the production of TDA by these isolates, the bioactive compounds extracted from the *Pseudovibrio* isolates were analysed via UHPLC-DAD-qTOFMS. This analysis confirmed the presence of TDA in the extract based on an identical retention time as an authentic reference standard, and the unique accurate mass of the [M + H]^+^ ion (212.9674 ± 0.005) as well as correct isotopic pattern of C_8_H_4_O_3_S_2_ (Figure 2b), unambiguously identifying TDA.

### 2.3. TDA-Producing Isolate W74 has Limited Activity against Marine Isolates

Having established that the TDA-producing isolates were active against a range of clinical and fish pathogens, we sought to investigate if TDA had an inhibitory effect against bacteria isolated from a similar environment or whether these bacteria would have some level of resistance against TDA, due to their close association. Therefore, the antimicrobial activity of W74 was tested against a range of marine bacteria isolated from 2 deep sea sponges, namely *Hexactinellida* and *Poecillastra* species. W74 was spot-plated and overlaid with 136 different marine isolates to screen for tolerance against TDA. Of the 136 isolates, 126 (93%) displayed tolerance to TDA. Tolerant species included *Psychrobacter*, *Alteromonas*, *Salinibacter*, *Alcanivorax*, *Flavobacterium* and *Micrococcus* species. Strains displaying sensitivity towards TDA included *Staphlococcus* species, as well as species of the genera *Idiomarina* and *Rhodococcus*.

The tolerant strains were subsequently tested for TDA production. No brown pigment production was observed in the TDA resistant marine isolates grown for 72 h from a starting OD_600_ of 0.01, suggesting that the isolates tested were not TDA producers. For confirmation of this, 12 resistant isolates were selected for compound extraction and tested for the presence of TDA using TLC spot-plate assays (Supplementary Table S3). All isolates tested negative for TDA production. These findings were interesting, as resistance to TDA has previously only been observed in TDA-producers alone [9,22,35]. A selection of 4 TDA sensitive isolates was also compound extracted and tested for TDA production, 3 of the 4 isolates tested negative for TDA production (Supplementary Table S3).

Interestingly, the *Pseudovibrio* strain W19, isolated from *Axinella dissimilis* and previously shown to have limited antimicrobial activity was found not to produce TDA using TLC analysis and was resistant to TDA extracted from W74. The resistance of non-producing W19 to TDA further suggests that the prevalence of TDA resistance mechanisms may be more widespread within marine communities than previously thought.

### 2.4. TDA Extracted from Pseudovibrio Displays Bioactivity against Cystic Fibrosis (CF) Clinical Isolates

Following the observed high levels of antimicrobial activity *Pseudovibrio* isolates displayed against a range of human pathogens, both previously [24] and this study, as well as the inhibitory effect displayed against the CF isolate *P. sputorum*, the effect of TDA extracted from the *Pseudovibrio* strain W74 was tested against a range of clinical isolates collected from the sputum of paediatric patients with CF from Cork University Hospital. TDA displayed antimicrobial activity against a number of CF isolates, namely *Bacillus cereus*, *Staphylococcus aureus*, *Staphylococcus epidermidis*, *Micrococcus luteus* and *Streptococcus haemolyticus.* Preventing chronic infection is essential in the maintenance of health in CF patients; hence it is of the outmost importance that suitable antibiotics are used to treat infection successfully. *S. aureus* has been shown to be the most common Gram positive organism found in the lungs of patients with cystic fibrosis, as well as being the second most persistent pathogen after *P. aeruginosa* [36]. Its sensitivity to TDA is all the more significant due to the fact that the above study revealed that *S. aureus* was not eliminated completely by oral antibiotics. The sensitivity of CF isolates to TDA was particularly interesting, as very little is known about the effect of TDA against clinical isolates. However, while a recent study by Neu *et al.* revealed that TDA has no toxicity when tested on the eukaryotic models *Artemia* sp. and *Caenorhabditis elegans* [30], further tests on animal models would be required before TDA could be considered as a suitable antibiotic for clinical use.

### 2.5. Genomic Pathway

**Figure 3:**
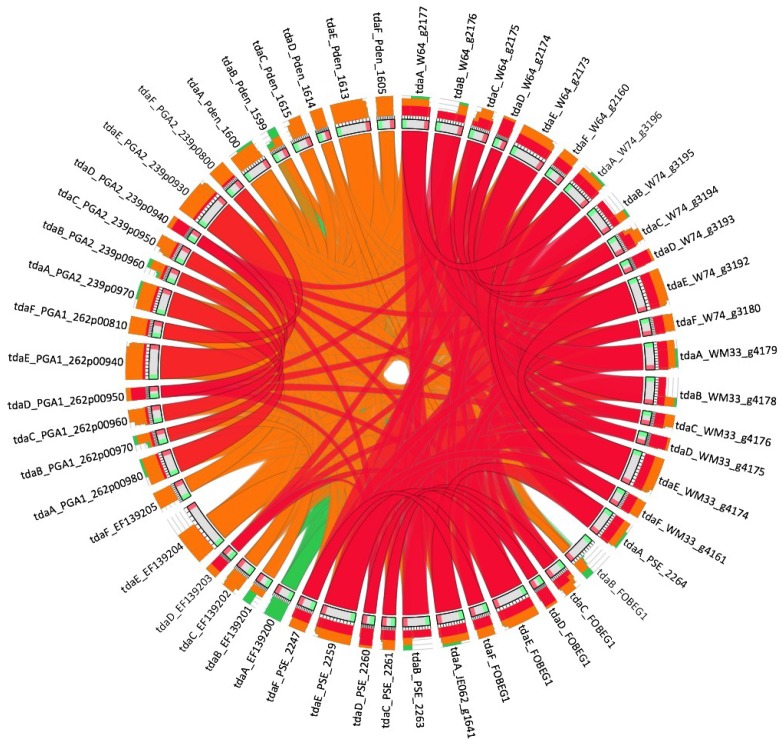
Comparative analysis of the TDA genes from *Pseudovibrio* compared to *Ruegeria* sp. TM1040. As expected, *Pseudovibrio* isolates exhibited the highest degree of sequence similarity to each other. Ribons represent the degree of sequence similarity with red being most similar and green signifying least similarity. Outer rings represent the coverage of sequence similarity in each gene as indicated by each coloured connecting line. See Supplementary Tables S4–S9 for the detailed similarity values between each pair of genes.

The TDA biosynthetic cluster was found in the draft genome sequences from the three marine isolates W64, W74 and WM33 after a NCBI BLAST+ 2.2.29 homology search using the TDA genes from *Ruegeria* sp. TM1040 [37] as query. The gene sequence identifier for each *tda*A-F homolog found in the draft genome sequences is reported in Table 2. All-vs-all homology comparison of the 6 genes involved in the TDA pathway for the different species [18,33,38,39] (Table 2) and the graphical representation was conducted by Circoletto [40] (Figure 3). The results from the all-vs-all comparative analysis indicate that the *tda* genes show a high degree of homology in all five marine *Pseudovibrio* isolates (>94% of similarity). Interestingly, of the 3 isolates tested in this study, we observed that W74 consistently produced higher levels of TDA when compared with other isolates under the same extraction conditions that cannot be fully explained by the sequence similarity. This higher level of TDA production was independent of any growth effects and may be due to differences in the regulation or production of TDA among marine isolates.

As the synthesis of TDA in Pseudovibrio isolates has not been characterised, we undertook to investigate the factors governing production of this important antimicrobial compound. Given that W74 produced the highest amounts of TDA under culture conditions, this isolate was selected for further analysis.

**Table 2:** Accession number for the six genes involved in the TDA pathway from the different species used in the comparative genomic analyses.

Strain	Accession Numbers of *tda* Genes					
	*tdaA*	*tdaB*	*tdaC*	*tdaD*	*tdaE*	*tdaF*
**W64**	W64_g2177	W64_g2176	W64_g2175	W64_g2174	W64_g2173	W64_g2160
**W74**	W74_g3196	W74_g3195	W74_g3194	W74_g3193	W74_g3192	W74_g3180
**WM33**	WM33_g4179	WM33_g4178	WM33_g4176	WM33_g4175	WM33_g4174	WM33_g4161
**FO-BEG1 [38]**	PSE_2264	PSE_2263	PSE_2261	PSE_2260	PSE_2259	PSE_2247
**JE062 [18]**	JE062_g1641	JE062_g1639	JE062_g1638	JE062_g1637	JE062_g1636	JE062_g1624
**TM1040 [37]**	EF139200	EF139201	EF139202	EF139203	EF139204	EF139205
***Phaeobacter gallaeciensis*** **DSM 17395 [33]**	PGA1_262p00980	PGA1_262p00970	PGA1_262p00960	PGA1_262p00950	PGA1_262p00940	PGA1_262p00810
***Phaeobacter gallaeciensis*** **2.10 [33]**	PGA2_239p0970	PGA2_239p0960	PGA2_239p0950	PGA2_239p0940	PGA2_239p0930	PGA2_239p0800
***Phaeobacter gallaeciensis*** **2.10 [39]**	Pden_1600	Pden_1599	Pden_1615	Pden_1614	Pden_1613	Pden_1605

### 2.6. TDA Expression Occurs during Logarithmic Growth

**Figure 4:**
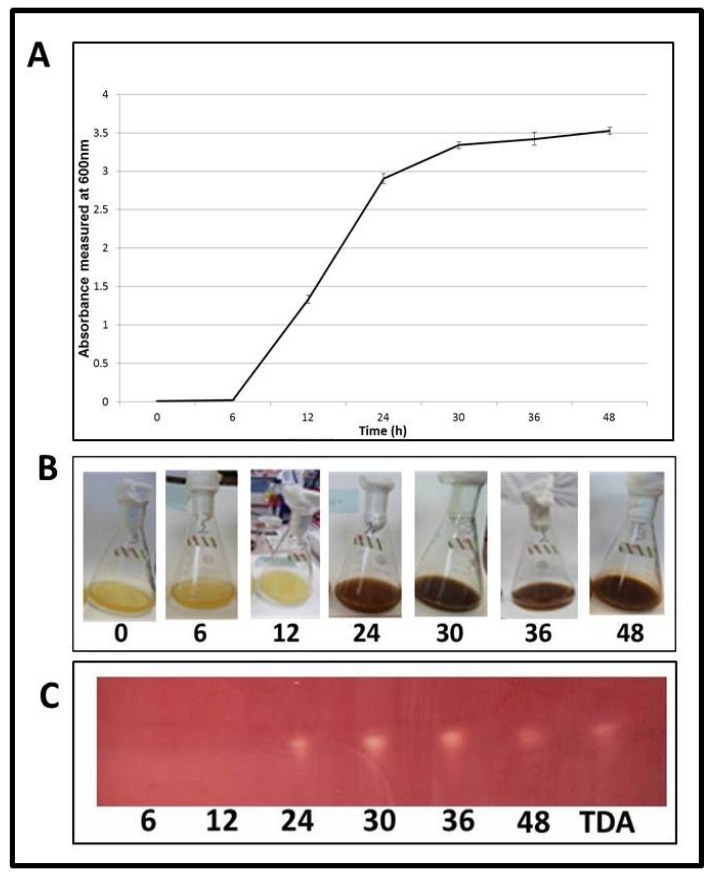
Timecourse experiment (0–48 h) for the production of TDA by *Pseudovibrio* strain W74. (**A**) Growth curve assay; (**B**) Pigment production; and (**C**) TLC-overlay assay of ethyl-acetate extracts obtained from W74 at the respective timepoints. TDA is included at a concentration of 0.02 μg/mL.

In order to determine at what point in the life cycle of *Pseudovibrio* TDA production occurs, W74 was grown under shaking conditions over a period of 48h. The growth was measured and production of the brown pigment was noted at 0, 6, 12, 24, 30, 36, and 48 h (Figure 4A,B). An aliquot of culture was collected for compound extraction at each time point, and bioactivity was tested by spotting extracted TDA on a TLC plate overlaid with the indicator strain *S. aureus* NCDO 949.

The colour change of W74 from opaque to brown occurred between 12 and 24 h, during the logarithmic stage of growth. From this point until the assay was completed at 48 h—*i.e.*, from logarithmic stage until late stationary phase—strong bioactivity was seen in the extract. Clear zones of inhibition of *S. aureus* NCDO 949 occurred where extracts were spotted (Figure 4C). This demonstrates that TDA production is initiated during the logarithmic growth phase. Microscopy of samples demonstrated that after 6 h (before colour change) cells were rod-shaped and existed in a planktonic form. However, subsequent to colour change (after 24 h), cells aggregated together in a rosette formation. This cell aggregation has previously been shown to be associated with the production of TDA in the *Silicibacter* strain TM1040 [37] and strains of *Ruegeria* and *Phaeobacter* [22,23,28] (Supplementary Figure S1). It is interesting to note that, although production of TDA occurred in both shaking and static cultures of W64 and W74, the amount of pigment production was much larger in the shaking cultures compared to those grown under static conditions. Furthermore, bacterial density was over 2-fold higher for isolates grown in shaking rather than static conditions. This is inconsistent with the findings of Bruhn and co-workers [35], who found that while cell density was up to 10-fold higher in shaking cultures, pigment production was only present in static cultures. It also contrasts markedly with the observations of Belas and colleagues [21,41] who demonstrated than TDA production was minimal in shaking cultures, and that of D’Alvise *et al.* [29] who showed that TDA expression in the *Roseobacter* clade species *Ruegeria mobilis* was only associated with attached or biofilm associated cells. This provides further evidence suggesting that differences exist in the regulation of TDA production in marine isolates.

### 2.7. Induction of TDA Genes in Pseudovibrio is Linked to Bioactivity

**Figure 5:**
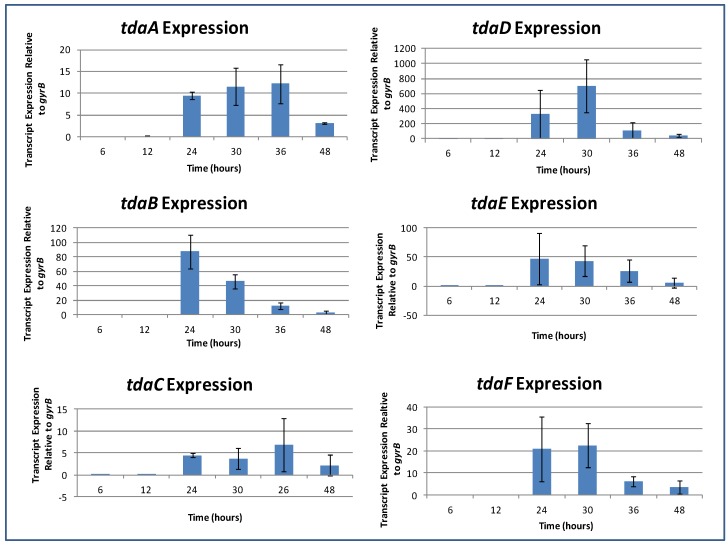
RT-PCR Data. *TdaA* and *tdaB* genes involved in the production of TDA were expressed at 24 h—directly correlating to both bioactivity and brown pigment production in cultures.

Gene expression profiles were generated by Real Time RT-PCR on samples of *Pseudovibrio* isolate W74 at 0, 6, 12, 24, 30, 36, and 48 h time points in order to examine the kinetics of expression governing TDA production. The expression of 6 genes involved in the TDA pathway—namely *tdaA*, -*B*, *-C*, *-D*, *-E* and *-F*, normalized to *gyrB*, was investigated. All genes were expressed by 24 h, with induction occurring almost simultaneously, demonstrating that the expression levels of genes involved in the TDA pathway was directly correlated to brown pigment formation and bioactivity. In all cases, expression levels dropped by 48 h—*i.e.*, late stationary phase (Figure 5). Induction and subsequent decline in expression of the genes encoding TDA strongly suggests that production of TDA is tightly controlled in *Pseudovibrio* isolates, as would be expected for an energy expensive process. A previous study has shown TDA production in *Phaeobacter gallaeciensis* (*inhibens*) to be under the control of quorum sensing 3OHC(10)-HSL (QS) [34], thus, the induction of TDA production in logarithmic growth, coupled with the reduction in gene expression in late stationary phase, suggested that quorum sensing may be involved in controlling TDA production in *Pseudovibrio* and warranted further investigation. Furthermore, studies have shown TDA to be auto induced, whereby TDA induced its own synthesis, in *Silicibacter* (*Ruegeria*) sp. and has been suggested to act as a QS signal [21]. Therefore, both auto-induction and QS-regulation of TDA were investigated in *Pseudovibrio* W74.

### 2.8. TDA is Not Autoinduced, or Induced by C10-AHL

**Figure 6:**
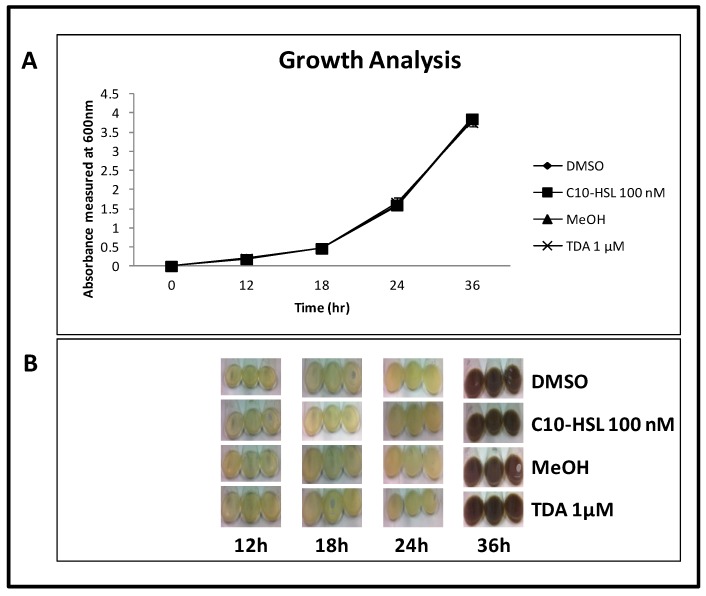
No evidence for autoinduction by TDA or early induction by QS compounds. (**a**) Growth curve of *Pseudovibrio* strain W74 grown in the presence of C10-HSL and TDA, along with the relative controls DMSO and MeOH; (**b**) Brown pigment production between 24 and 36 h. Up to 24 h, cultures remained a light beige colour. No difference is seen in the growth or colour of W74, indicating that TDA is not auto-induced, nor is it induced by C10-HSL.

In order to determine if TDA is auto-induced or induced by the QS molecule 3-OH-C10-HSL, W74 was grown in the presence or absence of TDA (1 μM), or 3-OH-C10-HSL (100 nM) respectively. Growth dynamics were identical in control and test cultures over 36 h (Figure 6A). In all cases brown pigment was produced at the same time interval (between 24 and 36 h) (Figure 6B). Previously, we had shown induction at 24 h, and the delay observed here is likely due to the presence of MeOH and DMSO in the media, resulting in delayed entry into logarithmic stage of growth. Cultures were compound extracted at each time point. All extracts—including a control of W74 grown in MB—showed TDA production at 36 h, suggesting that TDA production is not autoinduced or induced by 3-OH-C10-HSL.

Research by Geng and co-workers [37] revealed that TDA produced by *Silicibacter* (*Rugeria*) and *Pseudovibrio* species induced the transcription of *tdaC* in a TDA-producing *Ruegeria* sp. Autoinduction was not observed in our marine isolates, further evidence that production of this secondary metabolite is not uniform among marine isolates. However, further analysis and functional genomics studies will be required to determine the factors underlying this divergence.

## 3. Materials and Methods 

### 3.1. Growth Conditions

All *Pseudovibrio* strains were isolated from the sponges *Axinella dissimilis Polymastia boletiformis* and *Haliclona simulans* as previously described [16]. A selection of marine isolates from the UCC marine culture collection, previously isolated from the deep sea sponges of *Hexactinellida* and *Poecillastra* species, were used as indicator strains for TDA sensitivity assays. All marine sponge isolates were grown in the dark at 23 °C shaking at 200 rpm. Strains were grown on marine agar (MA) (Difco, Difco laboratories, Surray, KT8 2SE, UK), or SYP-SW agar (1% starch, 0.4% yeast extract, 0.2% peptone, 3.3% sea salt, 1.5% agar). Other bacterial isolates (indicator strains) were grown shaking at 200 rpm at 37 °C in LB (1% tryptone, 0.5% yeast extract, 0.5% salt).

### 3.2. Antimicrobial Spot-Plate Overlay Assay

Fifty microliters of *Pseudovibrio* strains grown overnight on Difco marine broth (MB, Difco laboratories, Surray, KT8 2SE, UK) were spotted at an OD_600_ of 0.2 onto MA or SYP-SW agar and incubated at 23 °C for 72 h. Bacterial indicator strains were grown overnight in LB and overlaid in 0.5% LB agar at an OD_600_ of 0.1. Zones of inhibition were measured after 24 h of incubation at 37 °C.

### 3.3. Compound Extraction

Compounds were extracted following a protocol adapted from Porsby *et al.*, [24]. Briefly, *Pseudovibrio* strains were inoculated from an overnight culture into 10ml MB at an OD_600_ of 0.01 and incubated shaking at 150 rpm at 23 °C for >72 h (or until brown colour change occurred). The culture was then centrifuged (4472× *g* @ RT) and filtered through a 0.2 micron filter (514-0061 VWR, VWR International Ltd., Dublin, Ireland). Equal volume ethyl acetate (10 mL) plus 100 μL formic acid were added to the cell-free supernatant and incubated shaking at 23 °C at 200 rpm for 10 min. Samples were centrifuged at 4472× *g* for 10 min. Subsequently, the upper layer was removed and vacuum concentrated at 50 °C until dry. The dried compound was then resuspended in relevant mobile phase, *i.e.*, 100 μL of MeOH for TLC and 200 μL of 85% acetonitrile/15% MilliQ water for UHPLC analysis. 

### 3.4. Thin Layer Chromotography (TLC)

Two microliters of extracts were spotted on a Silica gel TLC plate (60 F_254_, MERCK Millipore, Cork, Ireland). 2 μL of 10 μg/mL TDA (BVT-0152, BioViotica, Dransfeld, Germany), redissolved in MeOH was used as a control. The solvents used were dichloromethane and methanol at a ratio of 92.5:7.5, plus 0.15% acetic acid. When the front approached the top of the TLC plate, the plate was removed, dried and overlaid with 25 mL 0.5% LB agar containing bacterial indicator strains at an OD_600_ of 0.1, plus 5 mM 2,3,5-Triphenyltetrazolium Chloride (T8877, Sigma, St Louis, MO, USA) as a redox indicator of bacterial metabolism [42]. Plates were incubated for 24 h at 37 °C for pathogenic and laboratory indicator strains, and at 23 °C for marine isolates.

### 3.5. TDA Identification

*Pseudovibrio* extracts were redissolved in 200 μL 85% acetonitrile/15% MilliQ water. TDA standard solutions were diluted in 85% acetonitrile/15% MilliQ from a 1 mM TDA (BioViotica, Dransfeld, Germany) solution in dimethyl sulfoxide. UHPLC-DAD-qTOFMS analysis was conducted on an Agilent 1290 UHPLC coupled to an Agilent 6550 qTOF (Santa Clara, CA, USA) equipped with a dual electrospray source [43].

Separation was performed at 40 °C on a 2.1 mm ID, 50 mm, 1.8 μm Agilent Eclipse Plus C_18_ column [44] using a water-acetonitrile gradient solvent system, with both water and acetonitrile containing 20 mM formic acid. Using a flow of 0.8 mL/min, the gradient was started at 15% acetonitrile and increased to 60% acetonitrile within 1.8 min, then to 100% in 0.2 min, keeping this for 0.8 min, returning to 15% acetonitrile in 0.2 min, and equilibrating for the next sample in 1.5 min (total runtime 4.5 min). TDA was determined in ESI^+^ mode and quantified from its [M + H]^+^ ion 212.9674 ± 0.005 with the same retention time as the authentic standard (1.05 min). The TDA standard had a concentration of 0.01 mg/mL. Quantification was done using regression based on the peaks area in the Agilent MassHunter Quant 6.0 software (Agilent Technologies, Santa Clare, CA, USA).

### 3.6. Antimicrobial Spot-Plate Overlay Assay for Marine Sponge Isolates

The *Pseudovibrio* isolate W74 was spot-plated (5 μL) on MA at a starting OD_600_ of 0.2, and incubated at 23 °C for 72 h. Deep sea marine sponge isolates were grown overnight and inoculated into soft 0.5% marine agar at an OD_600_ nm of 0.1. This was overlaid onto W74 spot-plates and incubated at 23 °C. Zones of inhibition were measured after 24 h.

### 3.7. Pseudovibrio Induction Assay

*Pseudovibrio* strain W74 was inoculated from an overnight culture into 3 × 200 mL MB in 1 L flasks, at OD_600_ nm of 0.01 in the presence of 100 nM 3-OH-C10-HSL (University of Nottingham, Nottingham, UK), or 1 μM TDA, to test for the induction and autoinduction of TDA, respectively. W74 was also grown in the presence of 100 μL of both DMSO and MeOH, as C10-HSL and TDA were suspended in these respectively. W74 was also grown in marine broth as a control. Cultures were incubated at 200 rpm at 23 °C, and the OD_600_ of the cultures was measured at 12 h, 18 h, 24 h and 36 h. The colour of the cultures was also observed and noted at the above time points, at which times TDA was also extracted from the samples and analysed via TLC overlayed with *S. aureus* NCD0 949 as indicator strain.

### 3.8. RNA Isolation and cDNA Synthesis

*Pseudovibrio* strain W74 was grown shaking in 20 mL MB in a 100 mL flask at 23 °C for 48 h. Growth was measured at 6, 12, 24, 30, 36 and 48 h and a growth curve was plotted. At the above time points, 500 μL culture was collected and stored in 1 mL RNA protect. RNA was isolated as per the RNeasy RNA Extraction Kit protocol (Qiagen GmbH, Hilden, Germany). Isolated RNA was subsequently treated with DNase (0.1 volume 10× Turbo DNase) and incubated at 37 °C for 30 min to remove any potentially contaminating DNA. DNase was then inactivated using 0.1 volume DNase inactivation reagent. A 16S targeted PCR using 63F and 1387R primers was performed on the isolated RNA to test whether the DNase treatment had been successful [45]. RNA was converted to cDNA using AMV Reverse Transcriptase (Promega, Madison, WI, USA), RNasin (100 U/μL) (Promega, Madison, WI, USA), random primers (0.5 μg/μL) (Promega, Madison, WI, USA) and 10 mM dNTPs (Promega, Madison, WI, USA). A 16S targeted PCR was also performed using 63F and 1387R primers to ensure cDNA integrity.

### 3.9. RT-PCR

Quantitative RT-PCR analysis of six genes (tdaA-F) involved in TDA production was performed on W74 cDNA using primers outlined in Supplementary Table S9. Specific RT-PCR primers and probes were designed using the Universal ProbeLibrary assay center [46] for each gene based on the W74 genome sequence. Ten serial dilutions of the gDNA standards from 10^8^ to 10^4^ copies were prepared and 2 blanks were used as a control. Quantitative real-time PCR analysis of expression was carried out using the FastStart TaqMAN probe master kit (12747422, Roche, Basel, Switzerland). cDNA samples were diluted 1/10, and 5 μL was added to the following mixture: 12.5 μL Probemaster, 0.2 μL probe, 0.5μL forward primer, 0.5 μL reverse primer (Supplementary Table S9), 6.3 μL H_2_O. The mixture was incubated at 95 °C for 5 min, followed by 50 cycles at 95 °C for 40 s, 55 °C for 2 min, and 72 °C for 1 min. Quantitative real time-PCR signals were normalized to the constitutively expressed housekeeping gene *gyrB*. This gene was chosen on the basis that it is universally used for normalization of gene expression data in a broad spectrum of microbial species [47,48].

### 3.10. Genetic Analysis of Pseudovibrio TDA Genes

The 6 genes involved in the TDA pathway in *Ruegeria* sp. TM1040 [37] (Table 2) were used as a query to identify their homologs in the draft genomes of the three *Pseudovibrio sp* marine isolates W64, W74 and WM33 using NCBI BLAST+ 2.2.29 [49]. All-vs-all homology comparison of the 6 genes involved in the TDA pathway for the different species (Table 2) and the graphical representation was conducted by Circoletto [40].

## 4. Conclusions

In this study, we have characterised the production and spectrum of activity of TDA from marine sponge bacterial isolates, while also revealing an apparent lack of auto-induction or of QS induced early induction of TDA production in *Pseudovibrio*. In contrast to previous studies, tolerance to TDA, which was prevalent among marine sponge isolates, was not associated with native TDA production in these strains. TDA was shown to be active against a broad spectrum of human and fish pathogens, including *M. morganii* and *Pandoraeae sputonum*, both multidrug resistant opportunistic pathogens commonly associated with nosocomial infections [10,50,51,52,53]. TDA-producing *Pseudovibrio* isolates also displayed antimicrobial activity against *Salmonella enterica* ssp. *arizonae*, a serious albeit uncommon multidrug-resistant human pathogen which can cause life-threatening infections, usually in immunocompromised hosts [54,55,56]. *S. aureus*, one of the top three pathogens of blood stream infections [53], also displayed sensitivity to TDA produced by *Pseudovibrio* isolates. As the search for new antibiotics continues, TDA produced by marine sponge isolates has potential as a platform molecule for clinical development. Indeed, synthetic modification of the TDA framework resulting in analogues with enhanced antimicrobial activity has recently been reported [57], evidence that chemical modification of the compound can be achieved. Furthermore, initial toxicity studies have revealed that TDA is non-toxic in two eukaryotic models [30]. However, more detailed understanding of the molecular mechanisms governing TDA production is needed to underpin the necessary development process.

## Supplementary Files

Supplementary File 1
