# Supplementary material for: Characterisation of Non-Autoinducing Tropodithietic Acid (TDA) Production from Marine Sponge Pseudovibrio Species
Source: Mar Drugs. 2014 Dec 10;12(12):5960–78. doi: 10.3390/md12125960 (PMC4278212; doi:10.3390/md12125960)
Supplement: Supplementary File 1 [file marinedrugs-12-05960-s001.pdf]

Supplementary Information

Table S1. Antimicrobial Activity of the *Pseudovibrio* RAPD group representatives against the pathogen *S. aureus* NCDO 949, with *Pseudovibrio* isolates grown on MA *vs*. SYP-SW.

***Pseudovibrio* Isolate**	**MA**	**SYP-SW**
JIC5	++	<+
JIC6	++	-
JIC17	+	<+
W10	+	<+
W19	-	-
W62	++	+
W63	++	<+
W64	+++	<+
W65	++	+
W69	++	-
W71	+	<+
W74	++	<+
W85	++	<+
W78	+	<+
W89	+++	<+
W94	++	+
W96	+	<+
W99	++	<+
WM31	++	+
WM33	+	<+
WM34	++	<+
WM40	++	+
WM50	-	-
WC13	++	<+
WC15	+	-
WC21	++	<+
WC22	++	-
WC30	+	<+
WC32	++	-
WC41	++	+
WC43	++	<+
HC6	++	+
HMMA3	+	<+

Diameter Inhibition: <+ = <1 mm; + = ≥1 mm; ++ = ≥2 mm; +++ = ≥4 mm.

Table S2. Indicator strains used for testing bioactivity of marine isolates.

Strain	Description	Source/Reference
Yersinia ruckerri	Type strain	DSMZ [#]
Edwardsialla tarda	Type strain	DSMZ
Vibrio anguillarum LMG 4410	Type strain	DSMZ
Escherichia coli MUH 103	Clinical Isolate	MUH *
Escherichia coli NCIMB 15943	Type strain	MDCC UCC
Morganella morganii MUH 988	Clinical isolate	MUH
Salmonella Typhimurium LT2	Type strain	MDCC UCC
Salmonella Typhimurium C5369	Type strain	MDCC UCC
Pandoraea sputorum LMG18819	CF clinical Isolate	E. Caraher [§]
Salmonella arizonae	Type strain	Shinfield, UK/MDCC UCC
Staphylococcus aureus NCDO 949	Type strain	Shinfield, UK/MDCC UCC

[#] DSM Collection, Deutsche Sammlung von Mikroorganismen und Zellkulturen GmbH, Braunschweig, Germany; * Mercy University Hospital, Cork; [§] Centre of Microbial Host Interactions, ITT Dublin, Tallaght, Dublin 24, Ireland; ^ Microbiology Department Culture Collection, University College Cork.

Table S3. A selection of marine sponge isolates were compound extracted for TDA production. Both sensitive and tolerant isolates were tested for TDA production.

	Isolate	Nearest Neighbour	TDA Producer *
**Tolerant to TDA**	B98C31	*Vibrio*	×
	B98C32	*Micrococcus*	×
	B98C34a	*Salinibacterium amurskyense*	×
	B98C36	*Halomonas boliviensis*	×
	B98C38	*Maribacter*	×
	B98C49	*Alteromonas*	×
	B98C52	*Planococcus*	×
	B98C53	*Salinibacterium*	×
	B98C53b	*Psychrobacter*	×
	B98C56	*Alcanivorax*	×
	B98C69a	*Flavobacterium*	×
	B98S22a	*Oceanobacillus*	×
**Sensitive to TDA**	B98C7	*Staphlococcus saprophyticus*	×
	B98C30a	*Marinobacter maritimus*	√
	B98C44a	*Idiomarina*	×
	B98SM7	*Rhodococcus*	×

***** TDA production: × = no TDA; √ = yes TDA production.

Table S4. All-vs-all blastp similarity vaules between *tdaA* genes in the different species.

	**tdaA_EF139200**	**tdaA_JE062 _g1641**	**tdaA_PGA1 _262p00980**	**tdaA_PGA2 _239p0970**	**tdaA_PSE _2264**	**tdaA_Pden _1600**	**tdaA_W64 _g2177**	**tdaA_W74 _g3196**	**tdaA_WM33 _g4179**
tdaA_EF139200	100								
tdaA_JE062_g1641	48.7	100							
tdaA_PGA1_262p00980	42.29	65.88	100						
tdaA_PGA2_239p0970	43.28	66.22	98.65	100					
tdaA_PSE_2264	48.7	98.71	65.88	66.22	100				
tdaA_Pden_1600	41.12	53.12	54.73	55.4	53.12	100			
tdaA_W64_g2177	46.11	91.29	65.86	66.21	91.29	52.78	100		
tdaA_W74_g3196	46.11	91.29	65.86	66.21	91.29	52.78	100	100	
tdaA_WM33_g4179	46.11	90.97	65.86	66.21	90.97	51.32	99.03	99.03	100

Pseudovibrio sp. W64: tdaA_W64_g2177; *Pseudovibrio* sp. W74: tdaA_W74_g3196; *Pseudovibrio* sp. W33: tdaA_WM33_g4179; *Pseudovibrio* sp. FO-BEG1: tdaA_PSE_2264; *Pseudovibrio* sp. JE062: tdaA_JE062_g1641; Phaeobacter *gallaeciensis* DSM 17395: tdaA_PGA1_262p00980; *Phaeobacter gallaeciensis* 2.10: tdaA_PGA2_239p0970; *Ruegeria* sp. TM1040*:* tdaA_EF139200; *Paracoccus denitrificans* PD1222: tdaA_Pden_1600.

Table S5. All-vs-all blastp similarity vaules between *tdaB* genes in the different species.

	**tdaB_EF139201**	**tdaB_JE062 _g1639**	**tdaB_PGA1 _262p00970**	**tdaB_PGA2 _239p0960**	**tdaB_PSE _2263**	**tdaB_Pden _1599**	**tdaB_W64 _g2176**	**tdaB_W74 _g3195**	**tdaB_WM33 _g4178**
tdaB_EF139201	100								
tdaB_JE062_g1639	51.36	100							
tdaB_PGA1_262p00970	55.07	67.41	100						
tdaB_PGA2_239p0960	55.07	67.86	98.71	100					
tdaB_PSE_2263	50	94.83	66.52	66.96	100				
tdaB_Pden_1599	50.92	53.33	52.09	52.56	51.43	100			
tdaB_W64_g2176	51.85	83.17	61.81	61.81	82.21	50.79	100		
tdaB_W74_g3195	51.32	83.65	62.31	62.31	82.69	51.32	96.63	100	
tdaB_WM33_g4178	51.67	84.05	62.05	62.05	84.05	51.9	94.23	95.67	100

Pseudovibrio sp. W64: tdaB_W64_g2176; *Pseudovibrio* sp. W74: tdaB_W74_g3195; *Pseudovibrio* sp. W33: tdaB_WM33_g4178; *Pseudovibrio* sp. FO-BEG1: tdaB_PSE_2263; *Pseudovibrio* sp. JE062: tdaB_JE062_g1639; Phaeobacter *gallaeciensis* DSM 17395: tdaB_PGA1_262p00970; *Phaeobacter gallaeciensis* 2.10: tdaB_PGA2_239p0960; *Ruegeria* sp. TM1040*:* tdaB_EF139201; *Paracoccus denitrificans* PD1222: tdaB_Pden_1599.

Table S6. All-vs-all blastp similarity vaules between *tdaC* genes in the different species.

	**tdaC_EF139202**	**tdaC_JE062 _g1638**	**tdaC_PGA1 _262p00960**	**tdaC_PGA2 _239p0950**	**tdaC_PSE _2261**	**tdaC_Pden _1615**	**tdaC_W64 _g2175**	**tdaC_W74 _g3194**	**tdaC_WM33 _g4176**
tdaC_EF139202	100								
tdaC_JE062_g1638	57.72	100							
tdaC_PGA1_262p00960	60.12	71.76	100						
tdaC_PGA2_239p0950	60.12	71.76	99.5	100					
tdaC_PSE_2261	58.92	98.1	69.23	67.19	100				
tdaC_Pden_1615	55.09	60.31	64.33	64.33	60.23	100			
tdaC_W64_g2175	60	89.87	68.21	64.55	88.43	58.48	100		
tdaC_W74_g3194	60	89.87	68.21	64.55	88.43	58.48	100	100	
tdaC_WM33_g4176	59.06	89.87	67.94	67.94	89.24	57.25	98.1	98.1	100

Pseudovibrio sp. W64: tdaC_W64_g2175; *Pseudovibrio* sp. W74: tdaC_W74_g3194; *Pseudovibrio* sp. W33: tdaC_WM33_g4176; *Pseudovibrio* sp. FO-BEG1: tdaC_PSE_2261; *Pseudovibrio* sp. JE062: tdaC_JE062_g1638; Phaeobacter *gallaeciensis* DSM 17395: tdaC_PGA1_262p00960; *Phaeobacter gallaeciensis* 2.10: tdaC_PGA2_239p0950; *Ruegeria* sp. TM1040*:* tdaC_EF139202; *Paracoccus denitrificans* PD1222: tdaC_Pden_1615.

Table S7. All-vs-all blastp similarity vaules between *tdaD* genes in the different species.

	**tdaD_EF139203**	**tdaD_JE062 _g1637**	**tdaD_PGA1 _262p00950**	**tdaD_PGA2 _239p0940**	**tdaD_PSE _2260**	**tdaD_Pden _1614**	**tdaD_W64 _g2174**	**tdaD_W74 _g3193**	**tdaD_WM33 _g4175**
tdaD_EF139203	100								
tdaD_JE062_g1637	78.52	100							
tdaD_PGA1_262p00950	71.85	80.88	100						
tdaD_PGA2_239p0940	71.85	80.88	99.28	100					
tdaD_PSE_2260	78.52	99.31	80.88	80.88	100				
tdaD_Pden_1614	65.19	74.07	68.84	68.12	74.07	100			
tdaD_W64_g2174	77.04	93.06	80.43	80.43	92.36	72.59	100		
tdaD_W74_g3193	77.04	93.06	80.43	80.43	92.36	72.59	100	100	
tdaD_WM33_g4175	77.04	93.06	80.88	80.88	92.36	72.59	97.92	97.92	100

Pseudovibrio sp. W64: tdaD_W64_g2174; *Pseudovibrio* sp. W74: tdaD_W74_g3193; *Pseudovibrio* sp. W33: tdaD_WM33_g4175; *Pseudovibrio* sp. FO-BEG1: tdaD_PSE_2260; *Pseudovibrio* sp. JE062: tdaD_JE062_g1637; Phaeobacter *gallaeciensis* DSM 17395: tdaD_PGA1_262p00950; *Phaeobacter gallaeciensis* 2.10: tdaD_PGA2_239p0940; *Ruegeria* sp. TM1040*:* tdaD_EF139203; *Paracoccus denitrificans* PD1222: tdaD_Pden_1614.

Table S8. All-vs-all blastp similarity vaules between *tdaE* genes in the different species.

	**tdaE_EF139204**	**tdaE_JE062 _g1636**	**tdaE_PGA1 _262p00940**	**tdaE_PGA2 _239p0930**	**tdaE_PSE _2259**	**tdaE_Pden _1613**	**tdaE_W64 _g2173**	**tdaE_W74 _g3192**	**tdaE_WM33 _g4174**
tdaE_EF139204	100								
tdaE_JE062_g1636	69.05	100							
tdaE_PGA1_262p00940	66.86	68.34	100						
tdaE_PGA2_239p0930	67.14	68.84	97.73	100					
tdaE_PSE_2259	68.77	98.99	68.59	68.59	100				
tdaE_Pden_1613	63.71	64.29	64.94	65.19	64.29	100			
tdaE_W64_g2173	68.77	93.97	69.04	69.29	93.72	63.5	100		
tdaE_W74_g3192	68.48	93.47	69.04	69.29	93.47	63.5	99.25	100	
tdaE_WM33_g4174	68.48	93.72	69.04	69.29	93.72	63.5	99.5	99.75	100

Pseudovibrio sp. W64: tdaE_W64_g2173; *Pseudovibrio* sp. W74: tdaE_W74_g3192; *Pseudovibrio* sp. W33: tdaE_WM33_g4174; *Pseudovibrio* sp. FO-BEG1: tdaE_PSE_2259; *Pseudovibrio* sp. JE062: tdaE_JE062_g1636; Phaeobacter *gallaeciensis* DSM 17395: tdaE_PGA1_262p00940; *Phaeobacter gallaeciensis* 2.10: tdaE_PGA2_239p0930; *Ruegeria* sp. TM1040*:* tdaE_EF139204; *Paracoccus denitrificans* PD1222: tdaE_Pden_1613.

Table S9. All-vs-all blastp similarity vaules between *tdaF* genes in the different species.

	**tdaF_EF139205**	**tdaF_JE062 _g1624**	**tdaF_PGA1 _262p00810**	**tdaF_PGA2 _239p0800**	**tdaF_PSE _2247**	**tdaF_Pden _1605**	**tdaF_W64 _g2160**	**tdaF_W74 _g3180**	**tdaF_WM33 _g4161**
tdaF_EF139205	100								
tdaF_JE062_g1624	70.56	100							
tdaF_PGA1_262p00810	71.51	70.65	100						
tdaF_PGA2_239p0800	71.51	70.65	100	100					
tdaF_PSE_2247	70.56	99.5	70.65	70.65	100				
tdaF_Pden_1605	53.63	55.31	51.61	51.61	54.75	100			
tdaF_W64_g2160	70	96.98	70.11	70.11	96.48	54.75	100		
tdaF_W74_g3180	70	97.49	70.65	70.65	96.98	54.75	98.49	100	
tdaF_WM33_g4161	70.56	97.49	70.65	70.65	96.98	54.75	99.5	98.99	100

Pseudovibrio sp. W64: tdaF_W64_g2160; *Pseudovibrio* sp. W74: tdaF_W74_g3180; *Pseudovibrio* sp. W33: tdaF_WM33_g4161; *Pseudovibrio* sp. FO-BEG1: tdaF_PSE_2247; *Pseudovibrio* sp. JE062: tdaF_JE062_g1624; Phaeobacter *gallaeciensis* DSM 17395: tdaF_PGA1_262p00810; *Phaeobacter gallaeciensis* 2.10: tdaF_PGA2_239p0800; *Ruegeria* sp. TM1040*:* tdaF_EF139205; *Paracoccus denitrificans* PD1222: tdaF_Pden_1605.

Table S10. Genes amplified, primer sequences and probes used in RT-PCR experiments.

Gene	Primer Sequence (5′–3′)	Universal Probe Library Number
gyrB	aacgtcgacccctgaaaat<br>tggaatctgcaccgtattca	15
tdaA	gtcttgccggtagcctgac<br>caagcagattgctcacaacg	67
tdaB	ttatgtactgttcggcaccttg<br>cactcggagcaagaattgg	44
tdaC	gcaagtccgttttgctgaga<br>ccgatgtgctcgtaaataattg	144
tdaD	tttgagatgcagcatgaaatg<br>tgagtggaagctgcattgg	9
tdaE	gtttcggtctgacgctcctga<br>gaaggtctgagctcctga	161
tdaF	acacccaatgccatcaactt<br>ttatcggtggaccagtctttc	56

Figure S1. (**A**) 400× microscopy of W74 cells after 6 h of growth, before brown pigment production. Cells exist here in a planktonic form; (**B**) shows cell aggregation at 24 h, subsequent to colour change.

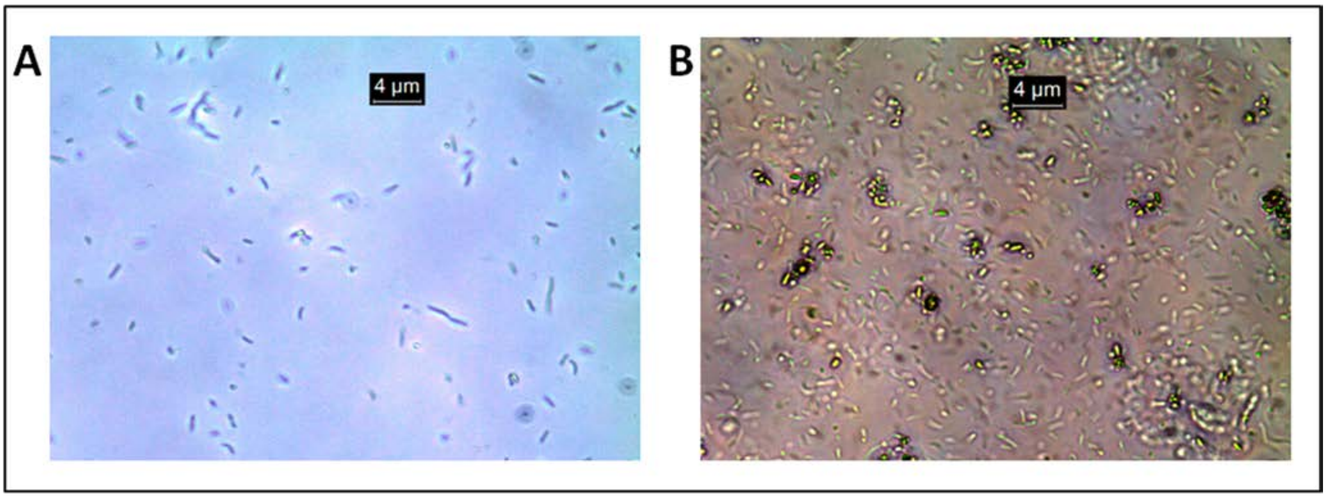

© 2014 by the authors; licensee MDPI, Basel, Switzerland. This article is an open access article distributed under the terms and conditions of the Creative Commons Attribution license (http://creativecommons.org/licenses/by/4.0/).
